# Neurocarta: aggregating and sharing disease-gene relations for the neurosciences

**DOI:** 10.1186/1471-2164-14-129

**Published:** 2013-02-26

**Authors:** Elodie Portales-Casamar, Carolyn Ch’ng, Frances Lui, Nicolas St-Georges, Anton Zoubarev, Artemis Y Lai, Mark Lee, Cathy Kwok, Willie Kwok, Luchia Tseng, Paul Pavlidis

**Affiliations:** 1 Centre for High-Throughput Biology and Department of Psychiatry, University of British Columbia, 2125 East Mall, Vancouver, BC V6T1Z4, Canada

**Keywords:** Phenotype, Genes, Knowledgebase, Brain development

## Abstract

**Background:**

Understanding the genetic basis of diseases is key to the development of better diagnoses and treatments. Unfortunately, only a small fraction of the existing data linking genes to phenotypes is available through online public resources and, when available, it is scattered across multiple access tools.

**Description:**

Neurocarta is a knowledgebase that consolidates information on genes and phenotypes across multiple resources and allows tracking and exploring of the associations. The system enables automatic and manual curation of evidence supporting each association, as well as user-enabled entry of their own annotations. Phenotypes are recorded using controlled vocabularies such as the Disease Ontology to facilitate computational inference and linking to external data sources. The gene-to-phenotype associations are filtered by stringent criteria to focus on the annotations most likely to be relevant. Neurocarta is constantly growing and currently holds more than 30,000 lines of evidence linking over 7,000 genes to 2,000 different phenotypes.

**Conclusions:**

Neurocarta is a one-stop shop for researchers looking for candidate genes for any disorder of interest. In Neurocarta, they can review the evidence linking genes to phenotypes and filter out the evidence they’re not interested in. In addition, researchers can enter their own annotations from their experiments and analyze them in the context of existing public annotations. Neurocarta’s in-depth annotation of neurodevelopmental disorders makes it a unique resource for neuroscientists working on brain development.

## Background

There is a tremendous amount of research focusing on understanding the genetic basis of disease. Studies use a wide range of strategies, including targeted gene approaches, genome-wide screens, and animal models. As such studies continue to proliferate and provide insights on specific disorders, it is important to integrate the information in order to make the best use of the data and increase the level of insight that can be gained from new studies. Knowledge that crosses studies and disorders can be used to perform meta-analyses, to uncover commonalities among conditions, and to tease apart the factors that contribute to phenotypes that make up a disorder. However, currently, information about the genetic and molecular basis of diseases is distributed among a range of specialized or generic data resources, hindering its optimal use [1]. Examples of more or less generic databases are Online Mendelian Inheritance in Man (OMIM) [2], the Rat Genome Database (RGD) [3], and the Comparative Toxicogenomics Database (CTD) [4]. While these different resources overlap in their disease coverage and data sources, they are also complementary in that each of the curation teams making the annotations has different criteria for inclusion and different biases. Other databases are dedicated to specific disorders, these include the Simons Foundation Autism Research Initiative Gene Database (SFARI Gene) for autism [5], PDGene for Parkinson’s disease [6], Alzgene for Alzheimer’s disease [7], MSGene for multiple sclerosis [8], ADHDgene for Attention Deficit Hyperactivity Disorder [9], and CADgene for Coronary Artery Disease [10].

The resource we describe was motivated by the establishment of a large Canadian research network “NeuroDevNet”, with the goal of translating knowledge into improved diagnosis, prevention and treatment of neurodevelopmental disorders [11,12]. To facilitate the design and interpretation of genetics studies, we recognized the need for a resource that captures existing information, but none of the resources mentioned above was sufficiently comprehensive. This was in part because genetic investigations of two of the disorders of interest to NeuroDevNet, Fetal Alcohol Spectrum Disorder (FASD) and Cerebral Palsy (CP) were not well covered by any existing database.

Neurocarta is an online resource focusing on the genetic basis of neurodevelopmental disorders. In addition to containing manually curated information on disorders of interest to neurodevelopmental researcher, Neurocarta aggregates data from multiple disease gene resources so that the neurodevelopmental annotations can be examined in the context of other disease annotations, providing a better understanding of how generic the function of the gene might be.

## Construction and content

### Database schema and implementation

**Figure 1:**
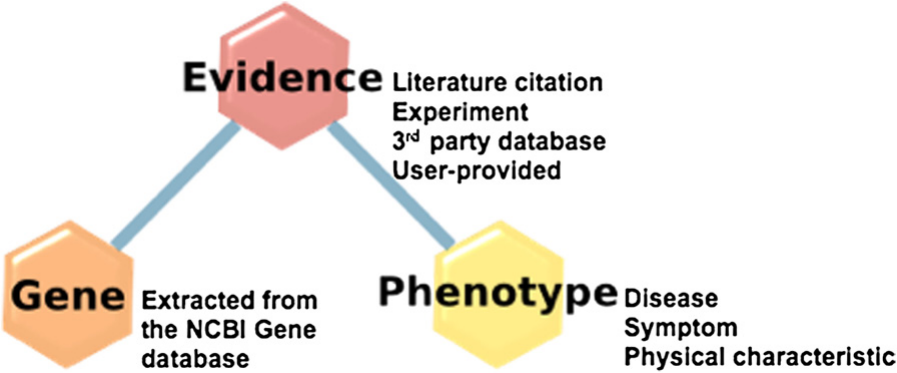
Gene-to-phenotype association data model in Neurocarta.

Neurocarta was developed as an extension of Gemma [13], a database and software system for the meta-analysis of functional genomics data. Figure 1 shows a simplified schematic of our data model used to capture information linking genes and phenotypes. The “Gene” information is retrieved automatically as part of the Gemma framework from the NCBI Gene database [14]. Gemma currently focuses on a set of selected species: human, mouse, rat, zebrafish, fly, worm, and yeast. The “Phenotype” information includes terms describing diseases, symptoms, and abnormal physical characteristics, drawn from three distinct ontologies: (i) Disease Ontology [15]; (ii) Human Phenotype Ontology [16]; and (iii) Mammalian Phenotype Ontology [17]. The “Evidence” corresponds to annotations linking a specific gene to a specific phenotype. This evidence can be of several types: (i) Literature (reference from PubMed [18]); (ii) Experimental (details about experimental design, from a published article or not); and (iii) User comment. Where possible, links are provided to the original source of the evidence (e.g., public database, review article), and can be defined as “positive” or “negative”, where “negative” means that the evidence shows that there is no association between a gene and a phenotype. All evidence annotations use standardized terminologies such as the Ontology for Biomedical Investigations [19] to facilitate users’ interpretation and enable computational analysis. Currently we do not attempt to capture information on the specific genetic variants associated with the disease as such information is frequently not readily available in computable form, making acquisition challenging.

Neurocarta benefits from the registration system implemented in Gemma [13] allowing users the option of registering and entering their own annotations. The annotations can be set to be either public or private. When private, the owner can decide whom to share them with, using a group-based authorization framework.

### Database content

**Figure 2:**
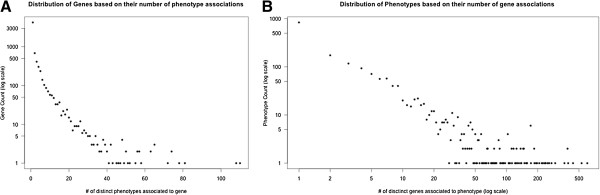
**Distribution of genes (2A) and phenotypes (2B) based on their number of distinct associations.** Each ontology term is considered a distinct phenotype regardless of its position in the ontology tree. Therefore, a gene will be counted as associated with two distinct phenotypes if different lines of evidence link it to a child term and its parent term.

Our database currently contains more than 30,000 lines of evidence linking over 7,000 genes to 2,000 different phenotypes (For detailed statistics, see http://www.chibi.ubc.ca/Gemma/neurocartaStatistics.html). Figure 2 shows the distribution of genes (2A) and phenotypes (2B) based on how many distinct associations they are a part of. Tables 1 and 2 detail the top ten genes and phenotypes, respectively, with the most distinct associations. The associations are derived from manual annotations from the literature and automatic annotations from public databases.

**Table 1:** Top ten genes with the most associated phenotypes

**Gene symbol**	**Gene name**	**NCBI ID**	**# of phenotypes**
TNF	tumor necrosis factor	7124	111
PTGS2	prostaglandin-endoperoxide synthase 2	5743	109
MMP9	matrix metallopeptidase 9	4318	82
IL6	interleukin 6	3569	79
PTEN	phosphatase and tensin homolog	5728	75
HLA-DRB1	major histocompatibility complex, class II, DR beta 1	3123	75
IL1B	interleukin 1, beta	3553	73
TP53	tumor protein p53	7157	66
MTHFR	methylenetetrahydrofolate reductase	4524	66
TGFB1	transforming growth factor, beta 1	7040	66

**Table 2:** Top ten phenotypes with the most associated genes

**Phenotype**	**Term URI**	**# of genes**
prostate cancer	http://purl.obolibrary.org/obo/DOID_10283	602
breast cancer	http://purl.obolibrary.org/obo/DOID_1612	531
hypertension	http://purl.obolibrary.org/obo/DOID_10763	439
autism spectrum disorder	http://purl.obolibrary.org/obo/DOID_0060041	394
type 2 diabetes mellitus	http://purl.obolibrary.org/obo/DOID_9352	389
asthma	http://purl.obolibrary.org/obo/DOID_2841	389
obesity	http://purl.obolibrary.org/obo/DOID_9970	363
peripheral nervous system disease	http://purl.obolibrary.org/obo/DOID_574	296
ovarian cancer	http://purl.obolibrary.org/obo/DOID_2394	273
Alzheimer’s disease	http://purl.obolibrary.org/obo/DOID_10652	259

### Data extraction from external sources

We have defined stringent criteria for automatic inclusion of data from external sources, with the goal of limiting the inclusion of unreliable data or information that we deem of limited utility to our target audience. In this section we provide details of procedures for each resource. As we are continuing to add resources to the system, information on the inclusion criteria and import procedures is also maintained on the Neurocarta website at http://gemma-doc.chibi.ubc.ca/neurocarta/data-sources.

OMIM [2]: The OMIM data files (morbidmap.txt and mim2gene.txt) are downloaded from the OMIM FTP site. We extract unique mappings between Phenotype MIM numbers and Gene MIM numbers from morbidmap.txt and map the genes to their NCBI identifiers in mim2gene.txt.

RGD [3]: The RGD Gene-Disease association files (homo_genes_rdo, mus_genes_rdo, rattus_genes_rdo) are downloaded from the RGD FTP site. Annotations with the following evidence codes are ignored: ISS (redundant across species), NAS (non-traceable author’s statements are debatable), and IEA (electronic annotations come from other sources, GAD for example) and we prefer to get these annotations directly from the source). Annotations without a PubMed reference are ignored as well.

CTD [4]: The CTD Gene-Disease association file (CTD_genes_diseases.tsv) is downloaded from the CTD website. We only consider curated annotations with Direct Evidence set to “marker/mechanism” or “therapeutic”, and at least one PubMed reference.

Disease-specific databases: The SFARI [5] annotation files (autism-gene-dataset.csv, gene-score.csv) are downloaded form the SFARI Gene website. Each PubMed reference is imported as separate literature evidence in Neurocarta, with the option of it being defined as “negative” whenever specified in the annotation file. The PDGene [6], AlzGene [7], and MSGene [8] “Top Results” are extracted from their respective websites. All three databases assess their results for their epidemiological credibility using two methods: (1) The HuGENet interim criteria for the cumulative assessment of genetic associations [20,21], and (2) Bayesian analyses [22,23]. Only meta-analysis results with P-values <0.00001 are considered. The “Hot gene list” from ADHDgene [9] is extracted from their website. This list includes all genes that have been identified in at least five independent studies. The ALSoD [24] top 20 genes are identified through the credibility score analysis provided on their website. The genes are ranked by the number of affected patients and by the number of mutations per gene, and the ranks are summed to determine the final rank for each gene. For the IDGene [25] and EpiGAD [26] databases, we wanted to extract more information than what was readily accessible through respective websites. We manually reviewed the genes listed in each database and used that information as a seed for targeted PubMed searches and manual curation of relevant publications.

### Disease mapping from external sources to Disease Ontology (DO) terminology

For the disorder-specific databases we use the corresponding appropriate terms in DO (e.g., “autism spectrum disorder” for SFARI and “amyotrophic lateral sclerosis” for ALSoD). As described next, for other databases we used a combination of automatic and semi-automatic methods for mapping.

OMIM, RGD, and CTD: These three resources provide OMIM or MeSH terms that we mapped to DO terms as follows. First, we use the Xref mappings provided in the Human_DO.obo ontology file, which covers about 50% of the phenotype-gene mappings in these resources. For the remaining that use terms lacking a DO Xref, we use the NCBO Annotator Web service [27] followed by manual quality control to resolve partial matches, increasing coverage substantially. In total about 2/3 of the phenotype-gene associations present in OMIM, RGD, or CTD could be mapped to a DO term. This is due to non-disease terms that are listed in OMIM but not in DO (e.g., “Blood type”, “Ig levels”), and some disease terms missing from DO (mostly syndromic, e.g., TARP syndrome, Jawad syndrome), or missed mappings. We have notified the DO maintainers of these gaps and expect to eventually be able to import a greater fraction of these annotations into Neurocarta.

### Manual curation of the literature

While the Neurocarta framework is generic, our curation team is focusing on annotations relevant to our primary research interest, neurodevelopmental disorders. In-depth annotations have been produced on the following Disease Ontology terms (including respective children terms): (i) “Autism Spectrum Disorder” (ASD; DOID_0060041); (ii) “Cerebral Palsy” (CP; DOID_1969); (iii) “Fetal Alcohol Spectrum Disorder” (FASD; DOID_0050696); (iv) “Epilepsy” (DOID_1826); and (v) and “Intellectual disability” (DOID_1059). When necessary, phenotype descriptions were complemented with more descriptive Human or Mammalian Phenotype Ontology terms such as “Memory impairment” (HP_0002354), “EEG abnormality” (HP_0002353), or “decreased brain size” (MP_0000774). Curators review the literature using PubMed searches across all fields (that is, the default PubMed setting) using queries such as “epilepsy” AND “genetics”. We avoid making searches that are gene-centric, except as a secondary mechanism to find additional citations on a gene-phenotype relationship identified through initial screening. When possible, review papers are used to identify primary research papers, which are then curated as “Experimental Type Evidence”. The curators record details about the experiment using controlled vocabularies, categorized as (for example) “Bio Source”, “Experiment Design”, or “Developmental Stage”. The criterion for inclusion is an experimentally-supported statement linking the gene to the phenotype. The exception is genome-wide studies where the results were not yet confirmed by follow-up experiments. The curated papers involve a wide variety of experiments including both animal models and human studies. For the former, if the authors describe the animal model as a specific model for the disorder of interest, the curators associate the gene studied in the paper directly to the human disease. If the authors describe an endophenotype that is related to the disease, the gene is associated to the endophenotype only. In some cases, review papers are used as the source of the annotations instead of drilling down to the original research papers. In that case, it is curated as “Literature Type Evidence” with no details about the experiments. To help users navigate through the evidence, we are, when possible, associating phenotypes to genes in a species-specific way. So, for instance, if the evidence comes from an experiment done in rats, it will be linked in Neurocarta to the rat gene.

## Utility and discussion

### User interface

**Figure 3:**
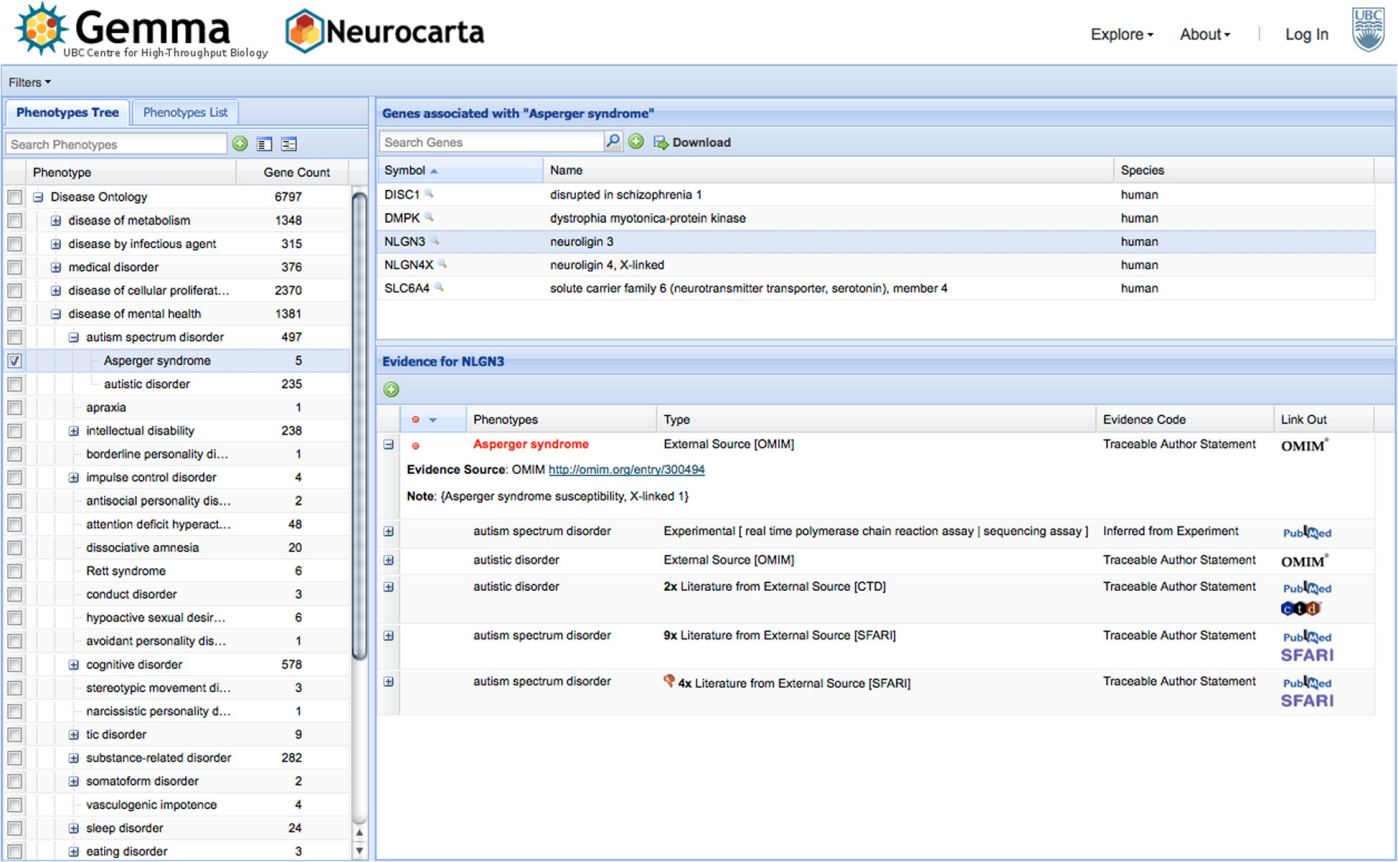
Neurocarta user interface.

Figure 3 shows the main Neurocarta user interface, which is divided into three panels. The left panel lists all phenotypes currently annotated in our system, displayed as a tree of terms in the ontologies, or as a simple list. By clicking on a checkbox next to the phenotype term, one or more phenotypes can be selected and it affects the display in the other two panels. The top-right panel shows the list of genes associated with the selected phenotype(s). If more than one phenotype are selected, only genes that are associated with all of the phenotypes are listed (i.e., the intersection of genes associated with each phenotype). A download button allows users to download the displayed gene lists. Once a gene of interest is selected, the bottom-right panel shows the list of evidence for all phenotype associations annotated for this gene, each row being expandable to provide more details. Evidence for the currently selected phenotype(s) and their children terms are highlighted in red. Evidence for other phenotypes associated with the gene are shown in black. Evidence inferred from an orthologous gene, as defined in the NCBI Homologene resource [14], are displayed in grey. Links are provided to the original source of the evidence when available. Users can filter the data displayed to restrict to a specific species, or to the annotations they have entered in Neurocarta.

### Use cases

Neurocarta was originally conceived to help researchers identify candidate genes that might be involved in their disorder of interest, based on existing knowledge, and put that information in the context of other phenotypes associated with the genes. Neurocarta allows users to extract the list of genes that have been associated to a specific disorder, look at the detail of the evidence, and apply further selection criteria. It can also be used to identify relevant literature pertaining to a gene or phenotype of interest. By aggregating data from multiple sources, we enable a global view of each gene’s involvement in diseases, facilitating the identification of genes specifically involved in one disorder versus genes involved in many disease processes. Such candidate gene lists can be used by researchers who perform genome-wide studies, helping them identifying the most likely candidates in their results. It can also inform more targeted approaches as to which gene to include in the study. Another unique aspect of Neurocarta is the ability that users have to enter their own annotations. This enables them to share unpublished results with collaborators as well as put their findings in the context of existing data and facilitate interpretation.

### Investigating gene-to-phenotype associations for neurodevelopmental disorders

**Figure 4:**
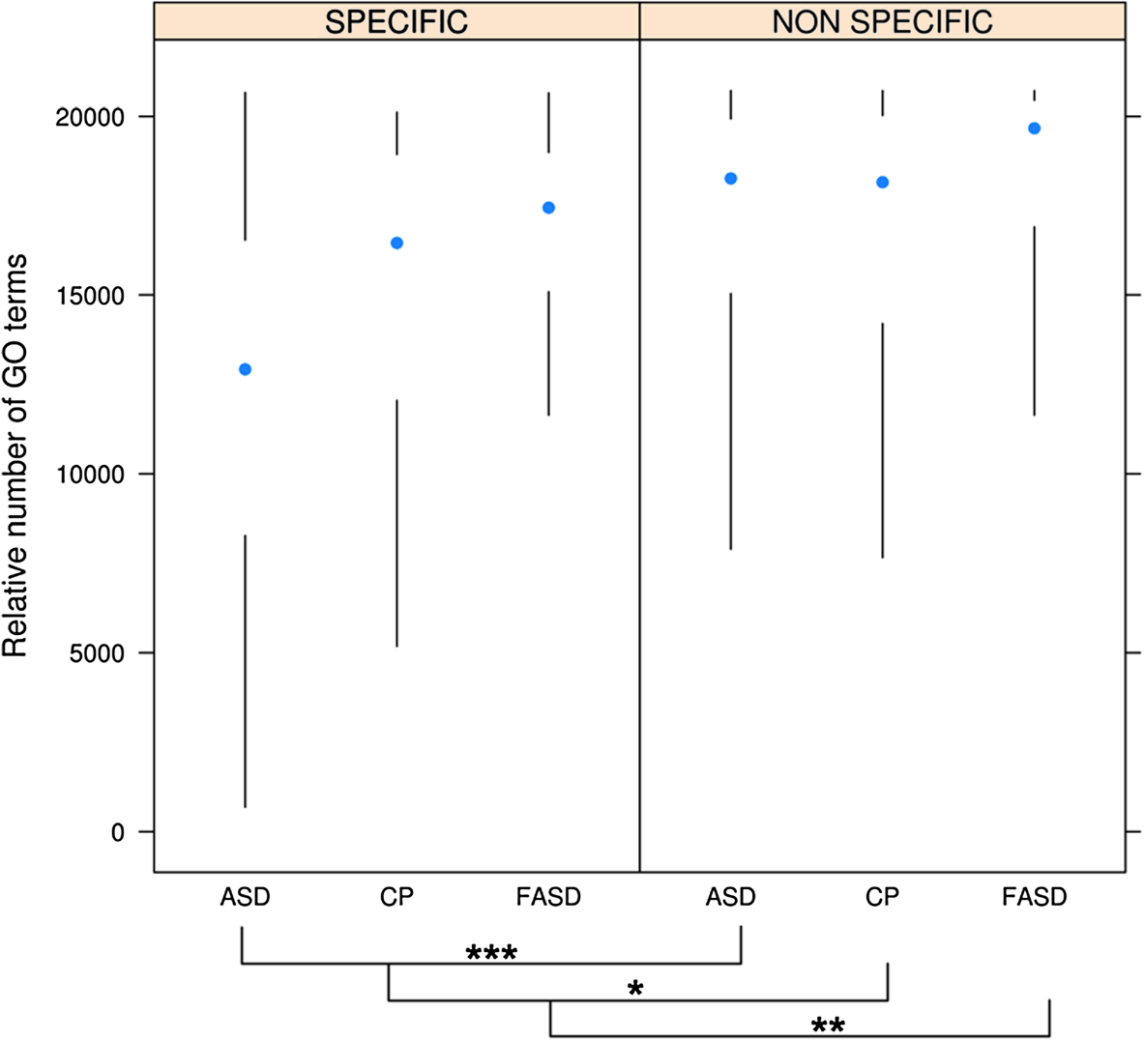
**Genes associated with multiple diseases in Neurocarta are more multifunctional than specific genes.** Mann–Whitney test: * P ≤ 0.1; ** P ≤ 0.01; *** P ≤ 0.001.

We gathered positive associations from Neurocarta (March 12, 2012). There were 14,983 unique gene-phenotype associations, consisting of many-to-many relationships between 4,560 genes and 1,555 phenotypes (Disease Ontology terms only). We decided to focus our analysis on neurodevelopmental disorders since we performed in-depth annotations on them. We categorized the genes based on which disease they were annotated for (ASD, CP, or FASD) and defined them as being “specific” if they were only associated with this one disease (Table 3). We observed that ASD has the largest fraction of specific genes. To try to better understand where the difference might come from, we decided to investigate whether or not biases might be present in the data. Previous work in our lab [28] showed that genes associated with diseases tend to be more “multifunctional” (i.e., they have more Gene Ontology (GO) [29] annotations). Therefore, we predicted that genes in Neurocarta would have a multifunctionality bias, and that the genes associated with multiple disorders would be even more multifunctional. Indeed, we found that genes in Neurocarta tend to be associated with a large number of GO annotations on average (aggregate multifunctionality score = 0.8, where 1.0 is the highest possible bias and 0.5 would be no bias). When we separated the genes specific to one disease versus the rest, we confirmed our hypothesis that genes associated with multiple disorders tend to be more multifunctional than specific genes (Figure 4; Mann–Whitney test, p-values: ASD = 2.6 × 10^-15^; FASD = 5.9 × 10 ^-3^; CP = 7.8 × 10^-2^). In addition, our results suggested that genes associated with FASD were more multifunctional than those associated with ASD or CP. We hypothesized that this might be due to the experimental approaches used to study FASD. Indeed, 98% of the FASD studies in Neurocarta are targeted (i.e., they use a candidate gene approach) against only 55% for ASD and 72% for CP, respectively. The multifunctionality bias in FASD candidate genes might thus be due to researchers choosing well-characterized genes for their studies rather than the genome-wide approaches mostly used in ASD research represented in the database. This might also explain the fact that, in Neurocarta, ASD has the largest fraction of specific genes, as mentioned above.

**Table 3:** Genes in Neurocarta associated with neurodevelopmental disorders

**Disease category**	**# of specific genes**	**Total genes**	**% of specific genes**
ASD	189	321	69.8
FASD	27	106	25.5
CP	23	124	22.1

ASD = Autism Spectrum Disorder; FASD = Fetal Alcohol Spectrum Disorder; CP = Cerebral Palsy.

### Comparison to similar existing resources

Several genotype to phenotype databases have been created with the idea of aggregating data from several sources in a common standardized online tool [1]. Some of existing tools rely entirely on OMIM annotations and only provide a more sophisticated portal to access data [29-31]. Others automatically aggregate data from a collection of resources, including OMIM, but they either focus only on human annotations [32], or on only one major phenotype database for a selection of model organisms [33]. Finally, some of the tools have been designed for human genetic association studies only and aggregate data from automatic or curated review of the literature [34-37]. Neurocarta is unique compared to these various initiatives in that it aggregates data from different organisms (human and animal models of diseases) and different kinds of studies (from genetic association to basic molecular experiments). It puts side-by-side data automatically extracted from public resources as well as manually curated from selected papers in the literature. All data goes through a review process where only the most reliable annotations are kept to reduce noise in the system. Finally, Neurocarta is the only publicly-available online tool that allows users to enter their own genotype to phenotype associations, share them with other users, and analyze them in the context of all existing annotations.

### Future development

We are planning several lines of improvements to Neurocarta’s data and software layers. Neurocarta currently includes very few data from genome-wide association studies because of the high rate of false positives that can arise from these data. We have included data from PDGene, AlzGene, and MSGene but only the top results that reached significance in their meta-analyses of the data. In the case of ADHDgene, we have decided to incorporate their Hot Gene list even though it was only based on the number of studies a gene was identified in. We are currently investigating different options to incorporate the most significant results from additional genetic association data from public resources such as GAD [35], GWASdb [36], or the GWAS catalog [37]. Another development that we feel will add value to Neurocarta is to incorporate automated Gemma differential expression analysis results. Neurocarta is part of Gemma but currently does not take a full advantage of this integration. In Gemma, gene expression datasets comparing control vs. disease cases are tagged and easily identifiable. We will apply differential expression analysis to these datasets using stringent thresholds to identify genes differentially expressed in specific diseases. We will then incorporate this analysis result as a new type of evidence linking genes to phenotypes in Neurocarta. Finally, a challenge in making the best use of the data is that different sources have different levels of evidence quality associated with them. For example, human geneticists would generally rate evidence from animal models as weak. Neurocarta does not directly capture such distinctions, so we are in the process of devising an evidence-rating scheme that will be used to automatically rank genes with respect to their strength of evidence in association with each disorder.

## Conclusions

Neurocarta is a new online resource linking genes to phenotypes. It brings together data from a wide variety of public resources and from manual curation of the literature. It is unique in that it allows users to enter their own annotations and keep them private if they wish to. In-depth annotations of genes involved in brain development disorders are available but Neurocarta is not restricted to a single disease. Instead, Neurocarta enables users to visualize all diseases their gene of interest might be associated with. This allows users not only to extract candidate gene lists from the system, but also to identify which of these genes are the most specific to their disorder of interest and to quickly find papers supporting these associations. Our analysis of the data in the context of neurodevelopmental disorders demonstrates that existing annotations linking genes to phenotypes are skewed to genes that are well known and involved in many biological functions. Neurocarta exposes this problem and makes it easier for researchers to focus their attention on more “specific” genes.

## Availability and requirements

Neurocarta is publicly available at http://neurocarta.chibi.ubc.ca.

## Abbreviations

ADHDgene: Attention deficit hyperactivity disorder gene database; AlzGene: Alzheimer’s disease gene database; ASD: Autism spectrum disorder; CP: Cerebral palsy; CTD: Comparative toxicogenomics database; FASD: Fetal alcohol spectrum disorder; GAD: Genetic association database; GO: Gene ontology; GWASdb: Genome-wide association study database; IEA: Inferred from electronic annotation; ISS: Inferred from sequence or structural similarity; MSGene: Multiple sclerosis gene database; NAS: Non-traceable author statement; NCBI: National Center for Biotechnology Information; OMIM: Online mendelian inheritance in man; PDGene: Parkinson’s disease gene database; RGD: Rat genome database; SFARI: Simons Foundation Autism Research Initiative.

## Competing interests

The authors declare that they have no competing interests.

## Authors’ contributions

EPC oversaw the development of Neurocarta and drafted the manuscript. CC conducted the data analysis. FL, NSG, and AZ developed Neurocarta. AL, ML, CK, WK, LT curated the data and tested the user interface. PP led the project and helped draft the manuscript. All authors read and approved the final manuscript.
